# Lipidomics Profiles Revealed Alterations in Patients With Meibomian Gland Dysfunction After Exposure to Intense Pulsed Light

**DOI:** 10.3389/fneur.2022.827544

**Published:** 2022-02-15

**Authors:** Hui Zhao, Shi-Nan Wu, Yi Shao, Dong Xiao, Li-Ying Tang, Zhe Cheng, Jie Peng

**Affiliations:** ^1^Department of Ophthalmology, Xinhua Hospital Affiliated to Shanghai Jiao Tong University School of Medicine, Shanghai, China; ^2^Department of Ophthalmology, Jiangxi Province Ocular Disease Clinical Research Center, The First Affiliated Hospital of Nanchang University, Nanchang, China; ^3^Department of Ophthalmology, Fujian Provincial Key Laboratory of Ophthalmology and Visual Science, School of Medicine, Xiamen University, Eye Institute of Xiamen University, Xiamen, China

**Keywords:** intense pulsed light, dry eye, disease markers, lipidomics, meibomian gland dysfunction

## Abstract

So far, intense pulsed light (IPL) has been widely used in the treatment of meibomian gland dysfunction (MGD), but there was still a lack of research on its specific mechanism. Determining whether there was a correlation between liposome changes and remission of clinical signs in patients with MGD treated with IPL was of great significance in the clinical evaluation of efficacy in patients with MGD. Our study enrolled the 10 healthy subjects and 26 adult patients, who were diagnosed with MGD and had not received any alternative treatments for at least 3 months. Each patient received a series of three treatments at 3-week intervals. The meibum was collected before the first treatment (T0) and the third treatment (T2). The significant changes in ocular surface parameters before and after IPL treatment were analyzed. The results showed that IPL significantly improved the symptoms of MGD, including ocular surface disease index (OSDI), tear breakup time (TBUT), redness of conjunctival (CR), corneal fluorescein staining (CF), the meibomian gland expressibility (MGE), and meibum quality (all *p* < 0.05). Lipidomics analysis of the meibum characterized the changes in lipid profiles induced by IPL. A total of 323 lipid species compounds were identified in the spectrum. A total of 41 lipid species were significantly different in patients with MGD (T0) vs. healthy controls. Following IPL treatment (T2), 24 lipid species were significantly different compared with T0: TG (10 lipid species), LPC (6 lipid species), OAHFA (4 lipid species), Cer (2 lipid species), SM (1 lipid species), and PE (1 lipid specie). Among these lipids, 4 of the lipids was a high correlation with TBUT, 5 was TH, 6 was CR, and 11 was meibum quality. In a ward, IPL treatment can achieve the therapeutic effect by changing the alternations of tear film lipids in patients with MGD. The changes in lipid expression profiles are potential indexes to evaluate the therapeutic effectiveness of IPL treatment or other treatments on MGD.

## Introduction

The meibomian gland (MG) is a lipid-rich tissue that is the main source of lipids for the tear film. Meibomian gland dysfunction (MGD) is a chronic, diffuse abnormality of the MGs, commonly characterized by terminal duct obstruction and/or qualitative or quantitative changes in glandular secretion. It may result in alteration of the tear film, symptoms of eye irritation, clinically apparent inflammation, and ocular surface disease (1). An epidemiological meta-analysis of MGD showed that the overall clinical prevalence rate was 35.8%. The prevalence rate of men was significantly higher than that of women (odds ratio = 1.24) (2). The MG is one of the components of the ocular surface microenvironment and its secretion plays an important role in tear film homeostasis (3). MGD was regarded as the leading cause of evaporative dry eye (DE) worldwide (4), especially related to the evaporative DE which is the most common ocular surface disease worldwide. Long-term MG lesions can cause inflammatory reactions in the ocular surface, which in turn lead to corresponding changes in the cornea and conjunctiva (5). In severe cases, visual acuity declines, resulting in a poor prognosis. At present, there is no uniform diagnostic standard for MGD (6). The widely accepted definition of “MGD” is a chronic diffuse abnormality of the MG, usually characterized by terminal duct obstruction and/or qualitative or quantitative changes in glandular secretions. This can lead to tear film changes, eye irritation, clinical inflammation, and ocular surface diseases. The commonly used diagnostic method is the generic diagnosis and systematic assessments, and its main inclusion indicators include the patient's symptoms, altered gland secretions, and changes in tear film (6, 7). Any of the above signs in combination with ocular symptoms are indicative of MGD. Due to the lack of symptomatic specificity assigned to MGD, it is often misdiagnosed and mistreated. These limitations in arriving at accurate diagnosis are prompting increasing attention by researchers in gaining insight into the pathophysiology underlying MGD.

The meibum, secretions of the MG, spreads onto the tear film to reduce the evaporation of the tear component and protects the eye from dust and microbial agents. The lipids secreted by MGs played a critical role in the homeostasis of the tear film. According to the related studies, the contents of triglycerides, cholesterol, and monounsaturated fatty acids in lipids in patients with MGD changed significantly (8). The meibum consists of an extremely complex mixture of lipids that is mainly composed of wax lipids, cholesterol lipids, free fatty acids, phospholipids, and a small amount of protein. More than 100 components of meibum lipids have been found, and hundreds of subtypes have not been identified thus far (9–11). Accurate evaluation of the changes in lipid expression profile is conducive to the treatment effect or disease progression of patients with MGD.

Numbers of therapeutics have been applied to treat MGD, such as antiinflammatory agents, immunosuppressors, tetracycline, lubricant ointments, artificial lubricants, eyelid hygiene, eyelid warming, massage (12), IPL (13), etc. However, there was not a standardized treatment for MGD. IPL is a noncoherent polychromatic light source with a broad wavelength spectrum of 500–1,200 nm (14, 15). As an established commercial technology, IPL treatment is broadly used in diseases involving facial rosacea. It has been shown that IPL is effective for the treatment of the eyelid sebaceous gland, also termed MG (16–18). Thus, IPL is a promising new therapy for MGD. Currently, IPL treatment is regarded as a more time-efficient and effective method to ameliorate the symptoms of DE (13). Although the efficacy and safety of IPL have been confirmed by ocular surface symptoms (18), the exact therapeutic effect of IPL on the MG remains unclear, especially the lipid metabolism. In this study, a comprehensive evaluation of clinical signs and changes in the lipid composition of meibum were conducted in patients with MGD after at least three exposures to IPL. A comparison of clinical assessment and alteration of the meibum were also conducted in patients treated with IPL and those without MGD. The results of this study would be helpful to understand the mechanisms of IPL in MGD treatment by providing insightful data.

## Patients and Methods

### Patients

Adult patients, who were diagnosed with MGD and had not received any alternative treatments for at least 3 months, were enrolled in the study. The inclusion criteria were as follows: diagnosis of MGD according to the Japanese MGD diagnostic criteria (9), including ocular symptoms, plugged gland orifices, vascularity of lid margins, irregularity of lid margins, and decreased meibum quality and quantity. The exclusion criteria were as follows: previous ocular surgery or trauma, blepharal dysraphism, history of blepharal and periorbital skin disease within 1 month prior to enrollment, acute inflammation, rheumatic immune systemic diseases, excessive sun exposure within 1 month prior to enrollment, history of herpes zoster infection, pregnancy, and use of photosensitive drugs or foods.

Informed consent was provided by all patients following an explanation of the nature and possible consequences of the study. This study was approved by the Institutional Review Board of the Xin Hua Hospital of Shanghai Jiao Tong University School of Medicine (Shanghai, China) and was registered with the Chinese Clinical Trial Registry prior to enrollment of the first patient. This study adhered to the tenets of the Declaration of Helsinki. All examiners were blinded to the treatment group. The study was enlisted in the clinical trial registry (trial registration no.: ChiCTR2000033454).

### IPL Treatment

Each patient received a series of three treatments at 3-week intervals. The patients had to use proparacaine hydrochloride eye drops (Benoxil, Santen Pharmaceutical Co., Ltd. Japan) at least 2 days prior to each treatment (19). A modular laser multiapplication platform (Quantum™, Lumenis, USA) was used to administer treatment to the periorbital area. During the IPL procedure, patients were required to wear opaque goggles, and the coolant was applied around the eyelids under both eyes. Makeup and contact lenses were removed before treatment. Depending on the patient tolerance, each subject in the patient group received 2–3 light pulses. The intensity of IPL treatment (14–16 J/cm^2^) depends on the Fitzpatrick skin with a 590-nm filter (19).

### Clinical Assessment

Visual acuity and intraocular pressure were recorded in the case-report form. The standard patient evaluation of eye dryness questionnaire was completed by each patient. Schirmer I test, corneal staining, meibum quality, and meibomian gland expressibility (MGE), lid margin abnormality, redness of conjunctival (CR), MG opening position, and corneal fluorescein staining (CF) were measured through slit-lamp microscopy. A noncontact infrared meibography system was used to assess tear breakup time (TBUT), MG dropout, and tear meniscus height (TH).

### Meibum Acquisition

The use of makeup, artificial tears, and cleaning the rim more than 3 h prior to the collection of the material was prohibited. After exposure to IPL, proparacaine hydrochloride eye drops were used again and the material was collected. The use of plastic products during sample preparation and experimentation was avoided to prevent contamination of the rouge. The steps performed for the collection of the materials were as follows: (1) the MG pressing method was used to squeeze out the secretions of the MGs with an MG expressor forceps – this is achieved by placing the thumb of one hand under the lower eyelid, squeezing the rim and rapidly pulling down, and squeezing out the secretion of the MG (the same method was used to remove the rouge from the upper eyelid); (2) a medical cotton swab was used to scrape the MG secretions, and the cotton swab was placed it in a cryotube and stored at −80°C until further analysis. The amount of rouge collected in this study was 1.77 ± 0.29 mg.

### One-Dimensional LC-MS/MS

Liquid chromatography separation using CSH C18 column (1.7 μm, 2.1 × 100 mm, waters) was selected to perform reverse-phase chromatography. The lipid of meibum was redissolved in 200 μL 90% isopropanol or acetonitrile then centrifuged at 14,000 x g for 15 min, and finally, 3 μL of the sample was selected for injection. Solvent A: acetonitrile–water (6:4, v/v) with 0.1% formic acid and 0.1 Mm ammonium formate. Solvent B: acetonitrile–isopropanol (1:9, v/v) with 0.1% formic acid and 0.1 Mm ammonium formate. The initial mobile phase was 30% solvent B at a flow rate of 300 μL/min. It was held for 2 min and then linearly increased up to 100% solvent B in 23 min, followed by equilibrating at 5% solvent B for 10 min. Mass spectra were acquired by Q-Exactive Plus in positive and negative modes, respectively. ESI parameters were optimized and preset for all measurements as follows: source temperature, 300°C; capillary temperature, 350°C, the ion spray voltage was set at 3,000 V, S-Lens RF Level was set at 50%, and the scan range of the instruments was set at m/z 200–1800.

“Lipid Search” is a search engine for the identification of lipid species based on MS/MS math. Lipid Search contains more than 30 lipid classes and more than 1,500,000 fragment ions in the database. Both mass tolerance for precursor and fragment were set to 5 ppm.

### Statistical Analysis

Data were analyzed using the SPSS version 22.0 (IBM Corp., Armonk, NY, USA) and R (version 4.0.2; package: corrplot version 0.90, psych version 2.1.9) software. Continuous intergroup variables were analyzed using an independent *t*-test, and pretreatment and continuous intragroup variables were analyzed with a paired *t*-test. Categorical intergroup variables were analyzed with the nonparametric Kruskal–Wallis test, and intragroup analysis of categorical variables was performed using the nonparametric Wilcoxon signed-rank test. Correlations between normally distributed values and nonnormally distributed values were analyzed with the linear Pearson's correlation coefficient and the Spearman's correlation coefficient, respectively. *p* < 0.05 denoted statistical significance.

## Results

### Patient Demographics

**Figure 1:**
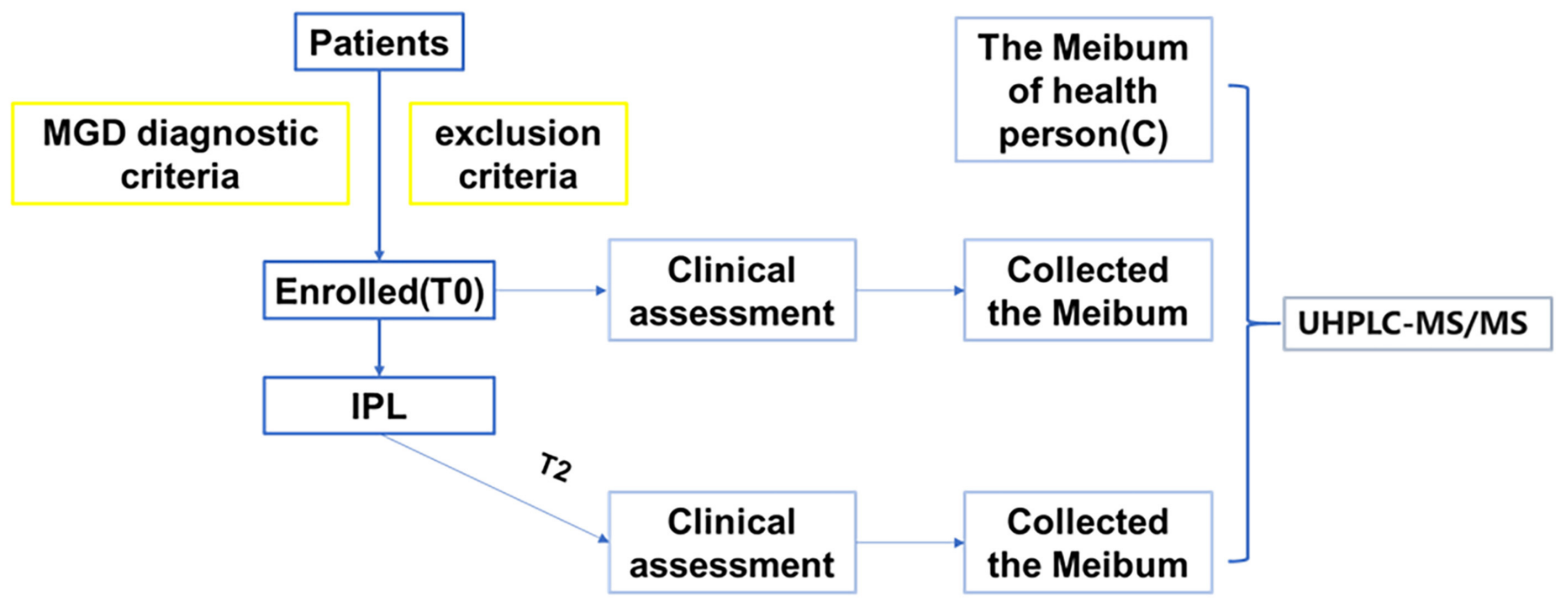
A working diagram of data processing.

A total of 26 patients' right eyes were enrolled in the study. We collected the meibum before the first treatment (T0) and the third treatment (T2). All enrolled patients completed the whole examination and treatment; there was no pain or any discomfort reported during the tests. All of the patients with MGD were asked to do the lipid analysis using LC-MS/MS. In addition, we also enrolled 10 volunteers who had no MGD diseases (the work plan is illustrated as follows) (Figure 1).

### Tear Film and Ocular Surface Analysis

Following treatment with IPL, the ocular surface disease index (OSDI) was markedly decreased from 43.10 ± 15.55 to 24.78 ± 12.85 (*p* < 0.01). At the same time, the TBUT (s) increased following treatment from 3.19 ± 1.13 to 5.62 ± 1.81 (*p* < 0.001). The CF of corneal epithelium integration and the CR inflammation were markedly improved. The CF score decreased from 1.00 ± 0.94 to 0.23 ± 0.43 (*p* < 0.001), and the CR scores reduced from 1.38 ± 0.62 to 0.58 ± 0.44 (*p* < 0.001), respectively (Table 1).

**Table 1:** Clinical parameters before and after treatment of IPL in MGD (*n* = 26).

**Clinical assessment**	**Before**	**After**	**T Value**	***P* Value**	
	**treatment**	**treatment**			
Age	48.89 ± 16.11	–	–	–	
Gender (Male/Female)	7/19	–	–	–	
OSDI	43.10 ± 15.55	24.78 ± 12.85	4.629	<0.001	^***^
TBUT(s)	3.19 ± 1.13	5.62 ± 1.81	−5.781	<0.001	^***^
TH	0.17 ± 0.08	0.21 ± 0.12	−1.386	0.172	
CR	1.38 ± 0.62	0.58 ± 0.44	5.399	<0.001	^***^
CF	1.00 ± 0.94	0.23 ± 0.43	3.801	<0.001	^***^
ML	1.04 ± 0.99	0.92 ± 1.01	0.417	0.679	
MG drop out	1.54 ± 0.86	1.54 ± 0.86	0.000	1	
MGE	1.31 ± 0.93	0.79 ± 0.53	2.474	0.017	^*^
Meibum quality	1.40 ± 0.85	0.73 ± 0.51	3.458	0.001	^**^

* *P < 0.01*,

** *P < 0.01*,

*** *P < 0.001; data showed as mean ± standard deviation or n*.

*OSDI, ocular surface disease index; TBUT, tear break-up time; TH, tear meniscus height; CR, redness of conjunctival inflammation; CF, corneal fluorescein staining; ML, marx line; MGE, meibomian gland expression*.

### Eyelid Margin Abnormalities

Our findings suggest that there was no statistically significant difference after the IPL treatment in terms of orifice abnormality and MG dropout (from 1.04 ± 0.99 to 0.92 ± 1.01, *p* = 0.679 and from 1.54 ± 0.86 to 1.54 ± 0.86, *p* = 1, respectively) (Table 1).

### MGE and Meibum Quality

The scores of MGE and meibum quality were used to evaluate the quality and expressibility of the meibum. The MGE score and meibum quality score were significantly decreased (from 1.31 ± 0.93 to 0.79 ± 0.53, *p* = 0.017 and from 1.40 ± 0.85 to 0.73 ± 0.51, *p* = 0.001, respectively) (Table 1).

### Lipid Classification and Kurtosis

**Figure 2:**
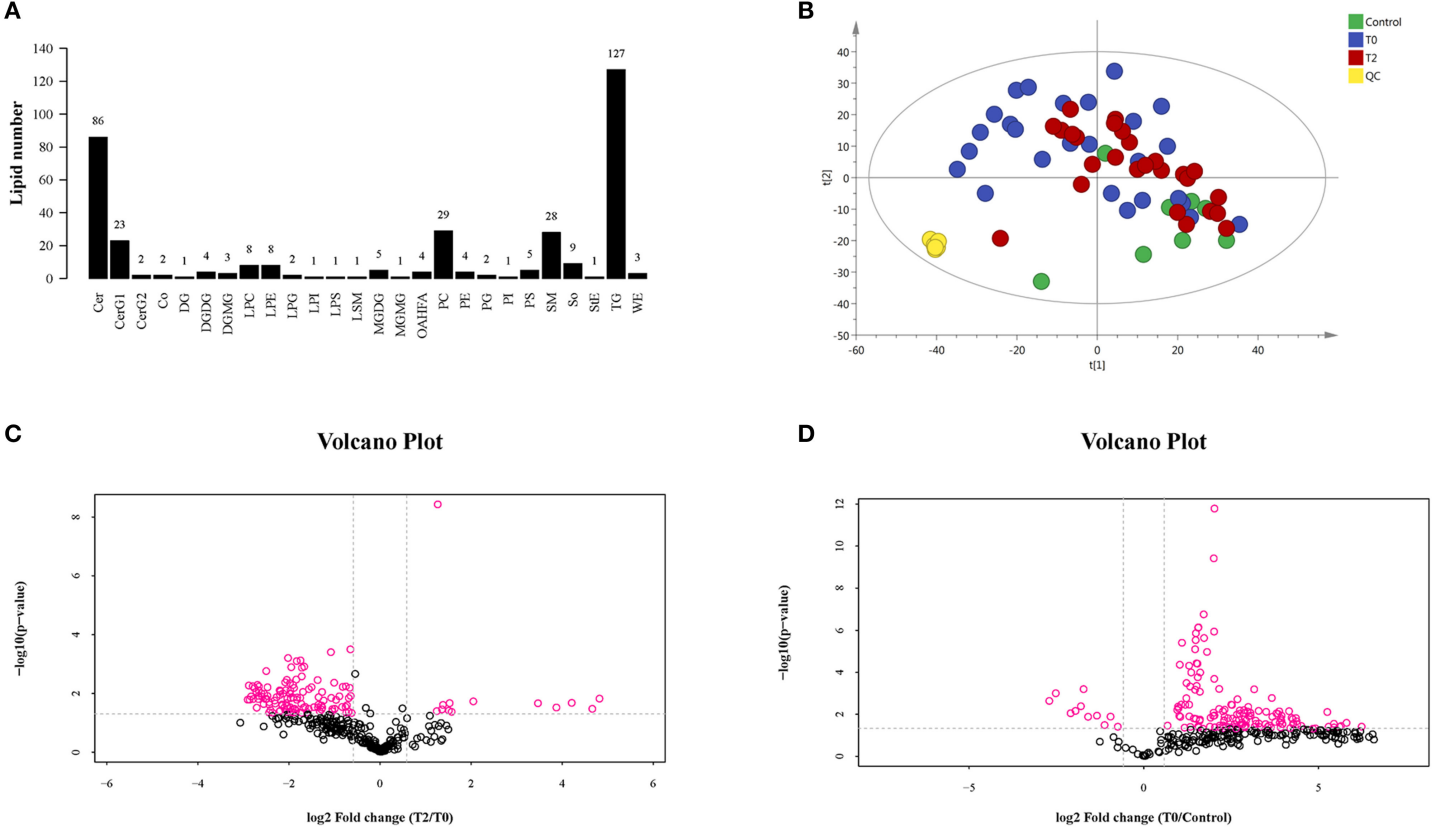
The LC-MS/MS analysis of human meibum in normal control (*n* = 10), before the first treatment of IPL (T0, *n* = 26) and the third treatment (T2, *n* = 26). **(A)** The identification of the lipid species. **(B)** PCA diagram among T0 group, T2 group, control group, and QC group. There was a significant trend distinction between T0 and control groups. **(C,D)**, respectively). The volcano plot of the lipids' quality of T2 vs. T0 **(C)** and T0 vs. control **(D)**.

A total of 323 lipid species were identified and most of them were rich in triglycerides (TG), ceramide (Cer), phosphatidylcholine (PC), sphingomyelin (SM), simple Glc series G1 (CerG1), sphingosine (SO), lysophosphatidylcholine (LPC), lysophosphatidylethanolamine (LPE), monogalactosyldiacylglycerol (MGDG), phosphatidylserine (PS), digalactosyldiacylglycerol (DGDG), and (O-acyl)-1-hydroxy fatty acid (OAHFA). More details can be seen in Figure 2A. The results of the principal component analysis (PCA) of the groups showed that there was an obvious distinguishing trend between T0 and control groups in lipid clusters (Figure 2B). When we compared the T2 to T0 and T0 to control, there were significant differences observed between the groups in lipid quality, as shown by the volcano plot (Figures 2C,D). Moreover, when we compared the T2 to T0, most of the different lipids were decreased in the former; when we compared the T0 to the control group, most of the different lipids were increased in T0.

**Figure 3:**
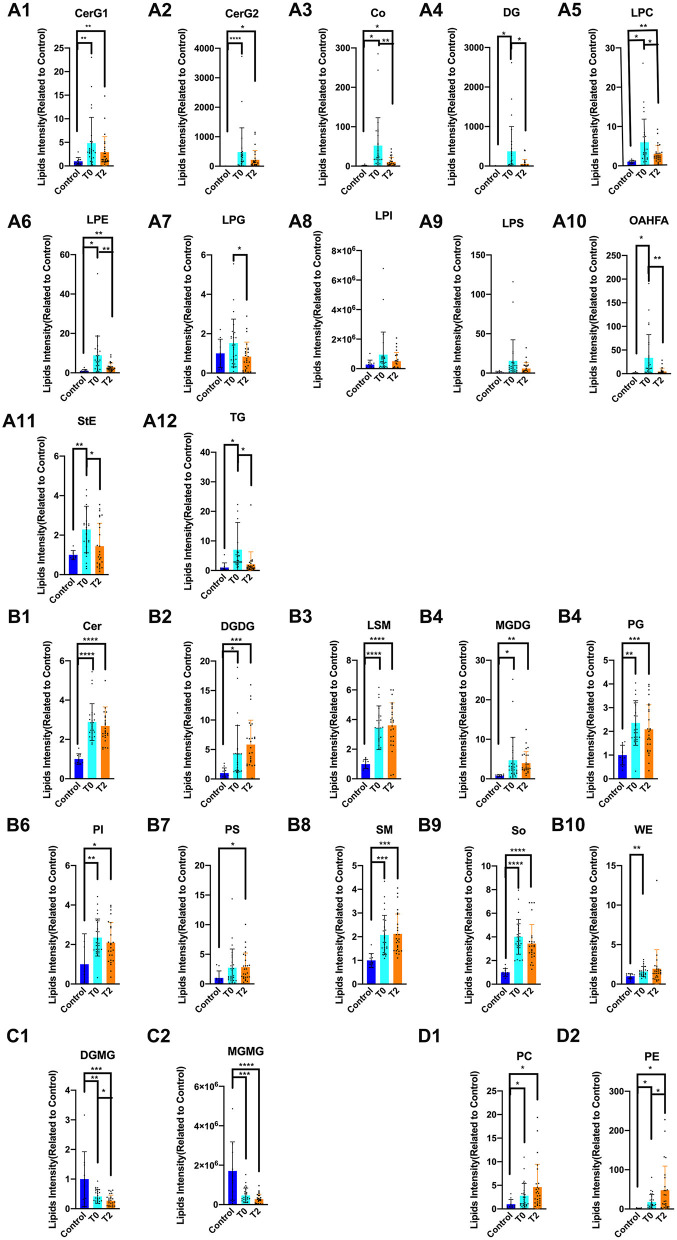
The alternation of lipid compound involved in IPL treatment. ^*^ *p* < 0.05, ^**^p < 0.01, ^***^ *p* < 0.001, ^****^p < 0.0001. Control (*n* = 10), T0 (*n* = 26), T2 (*n* = 26). **(A1–A12)** The increased lipids in T0 of CerG1, CerG2, Co, DG, LPC, LPE, LPG, LPI, LPS, OAHFA, StE, and TG were decreased after treatment of IPL(T2). **(B1–B10)** There was no significant difference in Cer, DGDG, LSM, MGDG, PG, PI, PS, and SM, So, WE that increased in T0 and T2. **(C1–C2)** The decreased lipids in T0 of DGMG and MGMG could be reversed by treatment of IPL(T2). **(D1–D2)** Even more, the PC and PE were higher than T0 in T2.

At the individual lipid compound level, the increased lipids in T0 (i.e., CerG1, CerG2, Co, DG, LPC, LPE, LPG, LPI, LPS, OAHFA, StE, and TG) were decreased following IPL treatment (T2) (Figures 3A1–A12). There were no significant differences in Cer, DGDG, LSM, MGDG, PG, PI, PS, SM, SO, and WE that increased in T0 and T2 (Figures 3B1–B10). The decrease in DGMG and MGMG in T0 could be reversed by IPL treatment (T2) (Figures 3C1,C2). Furthermore, the levels of PC and PE in T2 were higher than those measured in T0 (Figures 3D1,D2).

**Figure 4:**
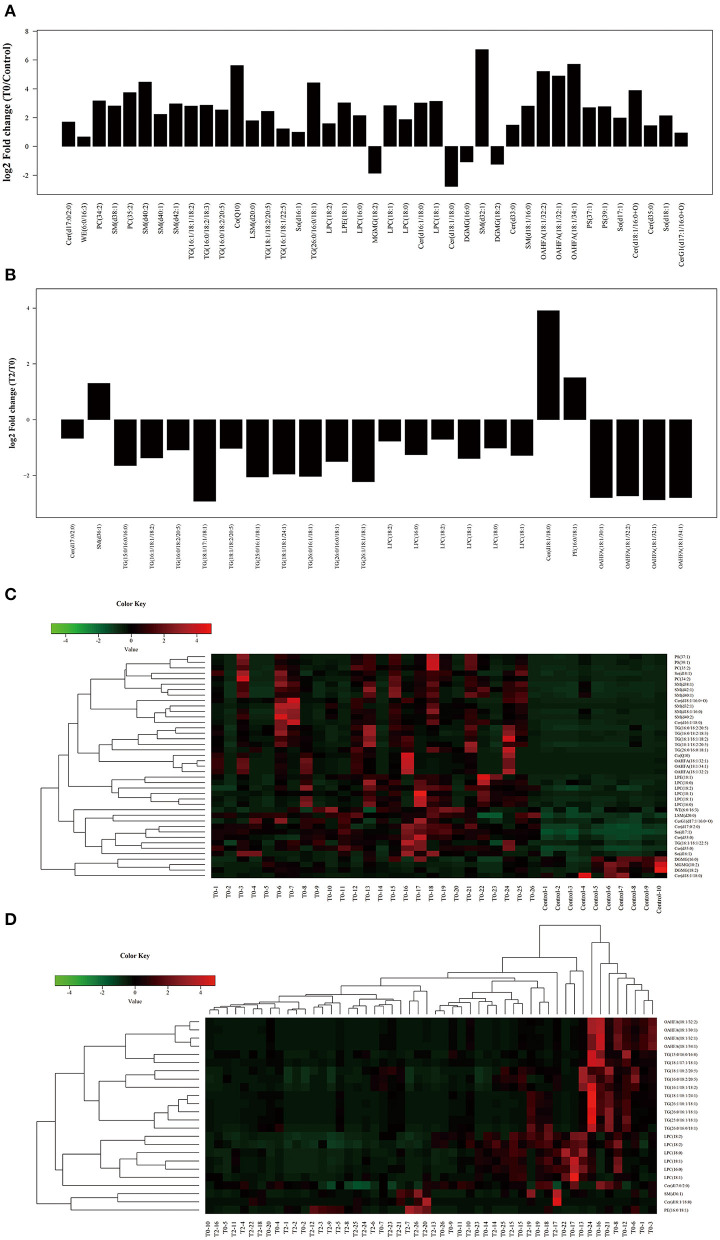
Significant different subclasses of lipids involved in IPL treatment. The fold change **(A)** and heatmap **(C)** of T0 vs. C; the fold change **(B)** and heatmap **(D)** of T2 vs. T0. Control (*n* = 10), T0 (*n* = 26), T2 (*n* = 26).

**Figure 5:**
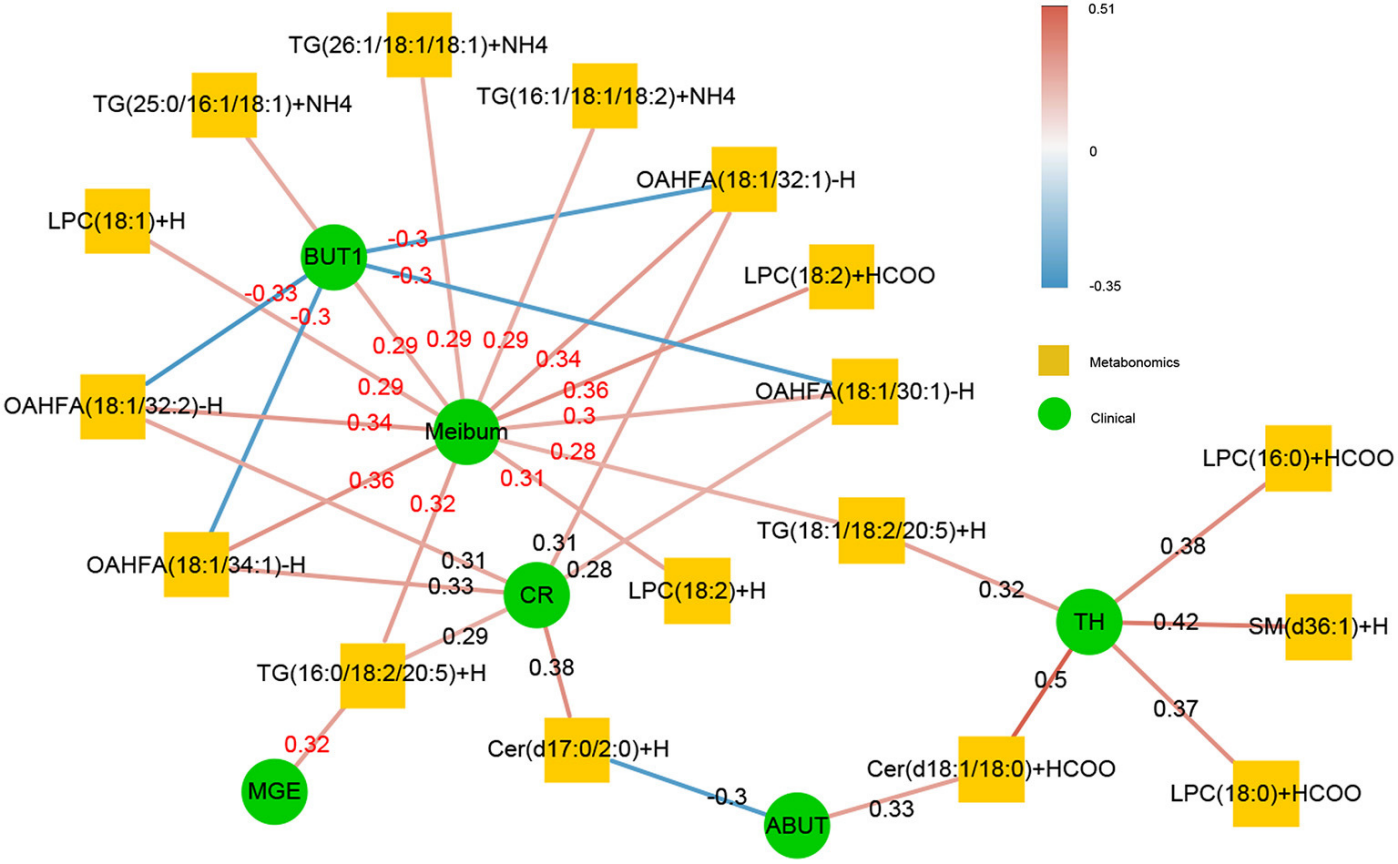
The correlation of significant subclasses of lipids with the clinical syndrome. The change in the lipid metabolic was highly related to the improvement in the clinical index. The correlation was rich in TBUT, CR, and meibum quality, and some lipids were crossrelated to them. The green in the middle represents the clinical index data, and the orange in the outer circle is the differential metabolic lipid. The darker the color of the line was, the stronger the correlation was, and the number on the line was the *r*-value of its correlation coefficient. The red line indicated that there was a positive correlation between the two, whereas the blue line represented a negative correlation between the two.

**Figure 6:**
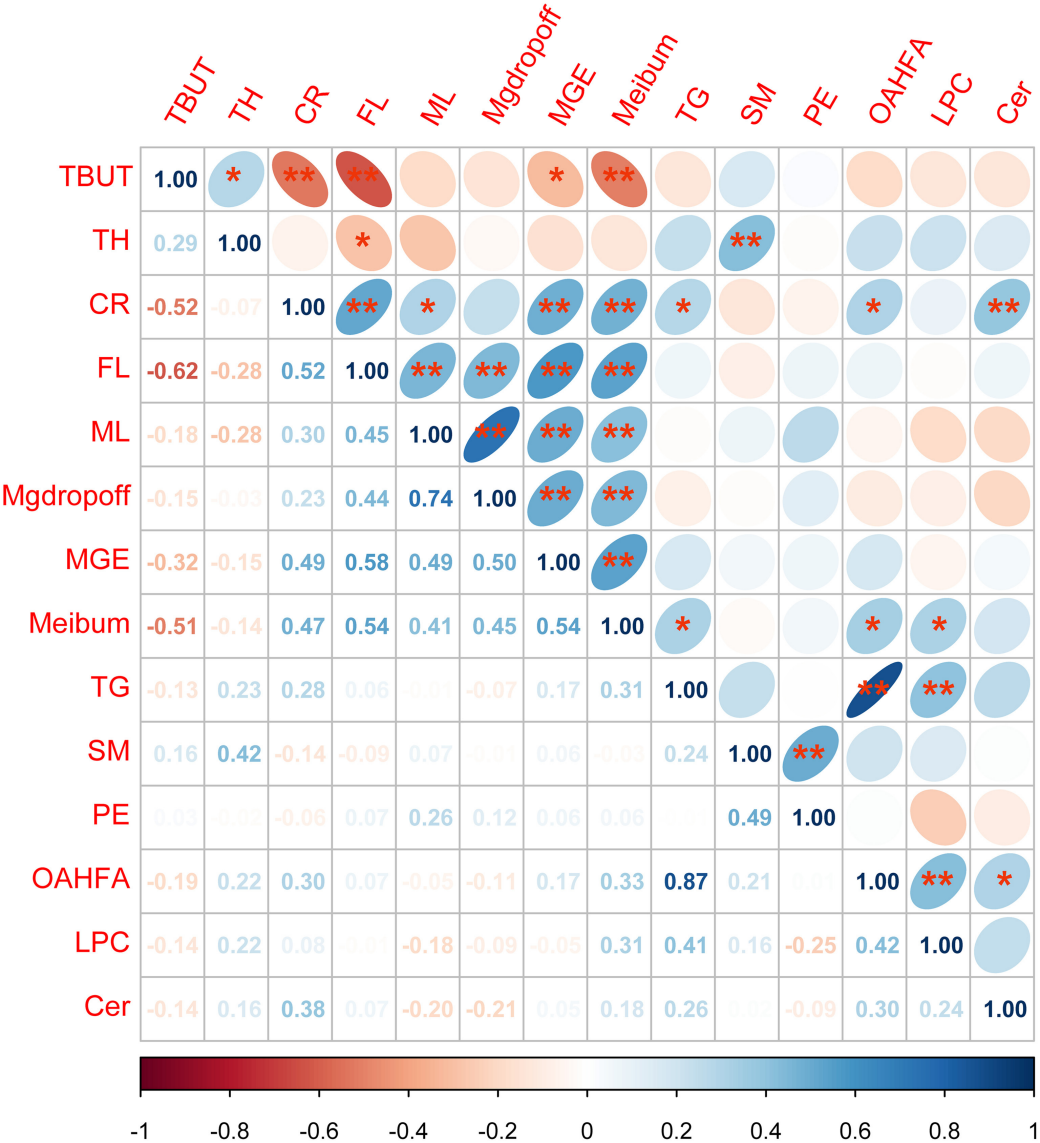
Analysis of the correlation between tear film lipid clusters and ophthalmic clinical indexes. *p < 0.05; **p < 0.01 Spearman's correlation analysis between all lipid cluster components and ophthalmic clinical indicators, the lower-left corner number is the *r*-value of correlation, the darker the color, the stronger the correlation. The ellipse in the upper right corner facing to the left is a positive correlation while facing to the right is a negative correlation. Blue represented a positive correlation between the two indicators, whereas red represented a negative correlation between the two indicators.

A total of 41 lipid species were significantly different in patients with MGD (T0) vs. healthy controls (Figure 4A). The difference between patients with MGD and controls included TG, SM, Cer, OAHFA, PC, DGMG, SO, PS, LPE, LSM, CerG1, CO, MGMG, and WE (Table 2). Following IPL treatment (T2), 24 lipid species were significantly different compared with T0: TG, LPC, OAHFA, Cer, SM, and PE (Table 3) (Figure 4B). The change in the lipid metabolic pattern was highly related to the improvement in the clinical index (Figures 5, 6; Table 5). The correlation was rich in TBUT, CR, and meibum quality, and some lipids were crossrelated to them. Notably, 4 lipid clusters were significantly increased in T0, whereas they were decreased following IPL treatment. The different lipids were OAHFA, LPC, TG, and Cer. More details can be seen in Table 4.

**Table 2:** Significant different subclasses of lipids of T0(*n* = 26) verse control (*n* = 10).

**LipidIon**	**Class**	**Calmz**	**IonFormula**	**RT-(min)**	**Fold Change**	**P-value**	**VIP**
SM(d32:1)+HCOO	SM	719.53	C38 H76 O8 N2 P1	8.743304	105.5802817	0.03955	1.27158
OAHFA(18:1/34:1)-H	OAHFA	785.74	C52 H97 O4	20.93825	52.16343297	0.03692	2.29603
Co(Q10)+NH4	Co	880.72	C59 H94 O4 N1	17.96348	49.2154925	0.03669	1.0111
OAHFA(18:1/32:2)-H	OAHFA	755.69	C50 H91 O4	18.67636	36.6853155	0.0444	2.04926
OAHFA(18:1/32:1)-H	OAHFA	757.71	C50 H93 O4	19.74312	29.63556255	0.04736	3.88388
SM(d40:2)+H	SM	785.65	C45 H90 O6 N2 P1	12.00555	21.94609662	0.03554	1.3621
TG(26:0/16:0/18:1)+Na	TG	995.9	C63 H120 O6 Na1	24.44222	21.32021673	0.02028	1.07358
Cer(d18:1/16:0+O)+H	Cer	554.51	C34 H68 O4 N1	10.187	14.87557017	0.01568	2.56661
PC(35:2)+H	PC	772.59	C43 H83 O8 N1 P1	10.84815	13.45758007	0.01122	1.40963
PC(34:2)+H	PC	758.57	C42 H81 O8 N1 P1	10.35652	9.044793221	0.02042	1.48086
LPC(18:1)+H	LPC	522.36	C26 H53 O7 N1 P1	2.829591	8.862523744	0.03903	1.58635
LPE(18:1)-H	LPE	478.29	C23 H45 O7 N1 P1	2.971951	8.196791624	0.0116	1.41347
Cer(d16:1/18:0)+HCOO	Cer	582.51	C35 H68 O5 N1	11.30124	8.162108672	0.00901	2.41341
SM(d42:1)+H	SM	815.7	C47 H96 O6 N2 P1	14.09983	7.808909475	0.00453	1.11772
TG(16:0/18:2/18:3)+Na	TG	875.71	C55 H96 O6 Na1	18.71938	7.301180438	0.0205	1.9029
LPC(18:1)+HCOO	LPC	566.35	C27 H53 O9 N1 P1	2.824801	7.164012181	0.00877	2.12477
SM(d38:1)+H	SM	759.64	C43 H88 O6 N2 P1	12.02927	7.023899481	0.0079	1.35097
SM(d18:1/16:0)+HCOO	SM	747.57	C40 H80 O8 N2 P1	9.977419	7.005964358	0.02223	2.06393
TG(16:1/18:1/18:2)+NH4	TG	872.77	C55 H102 O6 N1	19.68893	6.980513631	0.03026	1.53642
PS(39:1)-H	PS	830.59	C45 H85 O10 N1 P1	11.36284	6.821055558	0.03541	3.19895
PS(37:1)-H	PS	802.56	C43 H81 O10 N1 P1	10.3793	6.474225864	0.02929	1.56783
TG(16:0/18:2/20:5)+H	TG	877.73	C57 H97 O6	19.659	5.818026726	0.00493	2.28256
TG(18:1/18:2/20:5)+H	TG	903.74	C59 H99 O6	19.71597	5.444758925	0.00554	1.3387
SM(d40:1)+H	SM	787.67	C45 H92 O6 N2 P1	13.02854	4.693025458	0.01777	1.03408
LPC(16:0)+HCOO	LPC	540.33	C25 H51 O9 N1 P1	2.693533	4.427438179	0.0351	3.57942
So(d18:1)+H	So	300.29	C18 H38 O2 N1	2.422211	4.382913682	0.00565	2.80761
So(d17:1)+H	So	286.27	C17 H36 O2 N1	2.460979	3.940410295	3.91E-10	2.02106
LPC(18:0)+HCOO	LPC	568.36	C27 H55 O9 N1 P1	3.790138	3.64099302	0.03905	1.49809
LSM(d20:0)+H	LSM	495.39	C25 H56 O5 N2 P1	2.831587	3.444914731	1.08E-05	2.89417
Cer(d17:0/2:0)+H	Cer	330.3	C19 H40 O3 N1	1.913699	3.248423375	2.35E-06	1.85695
LPC(18:2)+H	LPC	520.34	C26 H51 O7 N1 P1	2.251227	2.988598263	0.00086	1.55151
Cer(d33:0)+H	Cer	526.52	C33 H68 O3 N1	9.731674	2.798888595	3.79E-05	1.15626
Cer(d35:0)+H	Cer	554.55	C35 H72 O3 N1	11.36469	2.720535667	8.07E-06	1.35036
TG(16:1/18:1/22:5)+H	TG	905.76	C59 H101 O6	15.60611	2.331510136	0.02129	1.30254
So(d16:1)+H	So	272.26	C16 H34 O2 N1	2.666427	1.985533979	0.01196	1.60794
CerG1(d17:1/16:0+O)+H	CerG1	702.55	C39 H76 O9 N1	9.514269	1.909466251	0.00627	1.18483
WE(6:0/16:3)+NH4	WE	352.32	H42 C22 O2 N1	3.006577	1.575320698	0.03507	1.08855
DGMG(16:0)+HCOO	DGMG	699.38	C32 H59 O16	2.678107	0.468996555	0.00695	1.61873
DGMG(18:2)+HCOO	DGMG	723.38	C34 H59 O16	2.225866	0.416667634	0.02013	2.13519
MGMG(18:2)+HCOO	MGMG	561.33	C28 H49 O11	2.625824	0.274625199	0.0003	1.38641
Cer(d18:1/18:0)+HCOO	Cer	610.54	C37 H72 O5 N1	12.32153	0.144934423	0.0018	1.9301

*Comparison of 41 lipid subgroups between MGD group and control group. MGD, meibomian gland dysfunction; T0, before treatment*.

**Table 3:** Significant different subclasses of lipids of T2 (*n* = 26) verse T0 (*n* = 26).

**LipidIon**	**Class**	**Calmz**	**IonFormula**	**RT-(min)**	**Fold Change**	***P*-value**	**VIP**
Cer(d18:1/18:0)+HCOO	Cer	610.5416	C37 H72 O5 N1	12.32153	15.03078	0.026831	3.35324
PE(16:0/18:1)-H	PE	716.5236	C39 H75 O8 N1 P1	11.40335	2.846096	0.021526	1.55677
SM(d36:1)+H	SM	731.6062	C41 H84 O6 N2 P1	11.06469	2.472157	0.033226	4.39805
Cer(d17:0/2:0)+H	Cer	330.3003	C19 H40 O3 N1	1.913699	0.628695	0.000317	1.16972
LPC(18:2)+HCOO	LPC	564.3307	C27 H51 O9 N1 P1	2.255877	0.61314	0.014431	1.04162
LPC(18:2)+H	LPC	520.3398	C26 H51 O7 N1 P1	2.251227	0.586953	0.005786	1.05027
LPC(18:0)+HCOO	LPC	568.362	C27 H55 O9 N1 P1	3.790138	0.494036	0.034213	1.26986
TG(18:1/18:2/20:5)+H	TG	903.7436	C59 H99 O6	19.71597	0.489044	0.006754	1.65819
TG(16:0/18:2/20:5)+H	TG	877.728	C57 H97 O6	19.659	0.470568	0.004624	1.1926
LPC(16:0)+HCOO	LPC	540.3307	C25 H51 O9 N1 P1	2.693533	0.418414	0.017479	3.33535
LPC(18:1)+H	LPC	522.3554	C26 H53 O7 N1 P1	2.829591	0.410042	0.03062	1.52317
TG(16:1/18:1/18:2)+NH4	TG	872.7702	C55 H102 O6 N1	19.68893	0.387629	0.013507	2.03988
LPC(18:1)+HCOO	LPC	566.3463	C27 H53 O9 N1 P1	2.824801	0.381895	0.003515	1.85389
TG(26:0/16:0/18:1)+Na	TG	995.8977	C63 H120 O6 Na1	24.44222	0.354542	0.018517	1.12173
TG(15:0/16:0/16:0)+NH4	TG	810.7545	C50 H100 O6 N1	21.12598	0.320491	0.027543	1.9067
TG(18:1/18:1/24:1)+NH4	TG	986.911	C63 H120 O6 N1	23.61661	0.259233	0.029449	1.11668
TG(26:0/16:1/18:1)+NH4	TG	988.9267	C63 H122 O6 N1	24.07609	0.244438	0.037562	1.01093
TG(25:0/16:1/18:1)+NH4	TG	974.911	C62 H120 O6 N1	23.85067	0.241359	0.035602	1.03776
TG(26:1/18:1/18:1)+NH4	TG	1014.942	C65 H124 O6 N1	24.10026	0.214039	0.027703	1.34062
OAHFA(18:1/32:2)-H	OAHFA	755.6923	C50 H91 O4	18.67636	0.150416	0.005079	2.50278
OAHFA(18:1/30:1)-H	OAHFA	729.6766	C48 H89 O4	18.52064	0.144494	0.007217	3.26419
OAHFA(18:1/34:1)-H	OAHFA	785.7392	C52 H97 O4	20.93825	0.144425	0.003703	2.80631
OAHFA(18:1/32:1)-H	OAHFA	757.7079	C50 H93 O4	19.74312	0.136615	0.004611	4.81245
TG(18:1/17:1/18:1)+NH4	TG	888.8015	C56 H106 O6 N1	21.16226	0.131759	0.016443	1.66444

*Comparison of 24 lipid subgroups between T2 group and T0 group. T0, before treatment; T2, after treatment*.

**Table 4:** Significant different subclasses of lipids of T0 (*n* = 26) verse control (*n* = 10) and T2 (*n* = 26) verse T0 (*n* = 26).

**LipidIon**	**Class**	**Calmz**	**IonFormula**	**RT-(min)**	**T0 vs. control**			**T2 vs. T0**		
					**Fold change**	***P*-value**	**VIP**	**Fold Change**	***P*-value**	**VIP**
OAHFA(18:1/34:1)-H	OAHFA	785.7392	C52 H97 O4	20.93825	52.16343297	0.036918	2.29603	0.14442499	0.003703	2.80631
OAHFA(18:1/32:1)-H	OAHFA	757.7079	C50 H93 O4	19.74312	29.63556255	0.047356	3.88388	0.136615378	0.004611	4.81245
OAHFA(18:1/32:2)-H	OAHFA	755.6923	C50 H91 O4	18.67636	36.6853155	0.044402	2.04926	0.150416143	0.005079	2.50278
LPC(18:1)+H	LPC	522.3554	C26 H53 O7 N1 P1	2.829591	8.862523744	0.039032	1.58635	0.410042149	0.03062	1.52317
LPC(18:0)+HCOO	LPC	568.362	C27 H55 O9 N1 P1	3.790138	3.64099302	0.039047	1.49809	0.494036433	0.034213	1.26986
LPC(18:1)+HCOO	LPC	566.3463	C27 H53 O9 N1 P1	2.824801	7.164012181	0.008767	2.12477	0.381895001	0.003515	1.85389
LPC(16:0)+HCOO	LPC	540.3307	C25 H51 O9 N1 P1	2.693533	4.427438179	0.035098	3.57942	0.418414154	0.017479	3.33535
LPC(18:2)+H	LPC	520.3398	C26 H51 O7 N1 P1	2.251227	2.988598263	0.000856	1.55151	0.586953112	0.005786	1.05027
TG(26:0/16:0/18:1)+Na	TG	995.8977	C63 H120 O6 Na1	24.44222	21.32021673	0.020276	1.07358	0.354541857	0.018517	1.12173
TG(18:1/18:2/20:5)+H	TG	903.7436	C59 H99 O6	19.71597	5.444758925	0.005544	1.3387	0.489043562	0.006754	1.65819
TG(16:0/18:2/20:5)+H	TG	877.728	C57 H97 O6	19.659	5.818026726	0.004927	2.28256	0.470568415	0.004624	1.1926
TG(16:1/18:1/18:2)+NH4	TG	872.7702	C55 H102 O6 N1	19.68893	6.980513631	0.030259	1.53642	0.387629187	0.013507	2.03988
Cer(d17:0/2:0)+H	Cer	330.3003	C19 H40 O3 N1	1.913699	3.248423375	2.35E-06	1.85695	0.628695394	0.000317	1.16972
Cer(d18:1/18:0)+HCOO	Cer	610.5416	C37 H72 O5 N1	12.32153	0.144934423	0.001796	1.9301	15.03077997	0.026831	3.35324

*A number of 13 lipids were significantly increased in T0, whereas they were decreased following IPL treatment. The different lipids were OAHFA(18:1/34:1)-H(C_52_H_97_O_4_), OAHFA(18:1/32:1)-H(C_50_H_93_O_4_), OAHFA(18:1/32:2)-H(C_50_H_91_O_4_), LPC(18:1)+H(C_26_H_53_O_7_N_1_P_1_), LPC(18:0)+HCOO(C_27_H_55_O_9_N_1_P_1_), LPC(18:1)+HCOO (C_27_H_53_O_9_N_1_P_1_), LPC(16:0)+HCOO(C_25_H_51_O_9_N_1_P_1_), LPC(18:2)+H(C_26_H_51_O_7_N1 P1), TG(26:0/16:0/18:1)+Na(C_63_H_120_O_6_Na_1_), TG(18:1/18:2/20:5)+H(C_59_H_99_O_6_), TG(16:0/18:2/20:5)+H(C_57_H_97_O_6_), TG(16:1/18:1/18:2)+NH4 (C_55_H_102_O_6_N_1_), and Cer(d17:0/2:0)+H(C19 H40 O3 N1)*.

*T0, before treatment; T2, after treatment*.

**Table 5:** Analysis of the correlation between lipid composition and ophthalmic indexes.

**Lipid**	**Ophthalmic**	**Correlation**	***P* value**
**composition**	**index**	**coefficient**	
Cer(d18:1/18:0)+HCOO	TH	0.504	0.000
SM(d36:1)+H	TH	0.422	0.002
LPC(16:0)+HCOO	TH	0.379	0.007
Cer(d17:0/2:0)+H	CR	0.376	0.007
LPC(18:0)+HCOO	TH	0.372	0.008
LPC(18:2)+HCOO	Meibum	0.363	0.010
OAHFA(18:1/34:1)-H	Meibum	0.357	0.011
OAHFA(18:1/32:2)-H	Meibum	0.342	0.015
OAHFA(18:1/32:1)-H	Meibum	0.339	0.016
OAHFA(18:1/32:2)-H	BUT1	−0.330	0.019
Cer(d18:1/18:0)+HCOO	ABUT	0.326	0.021
OAHFA(18:1/34:1)-H	CR	0.326	0.021
TG(16:0/18:2/20:5)+H	Meibum	0.324	0.022
TG(16:0/18:2/20:5)+H	MGE	0.323	0.022
TG(18:1/18:2/20:5)+H	TH	0.319	0.024
OAHFA(18:1/32:1)-H	CR	0.312	0.027
LPC(18:2)+H	Meibum	0.308	0.030
OAHFA(18:1/32:2)-H	CR	0.306	0.031
Cer(d17:0/2:0)+H	ABUT	−0.304	0.032
OAHFA(18:1/34:1)-H	BUT1	−0.304	0.032
OAHFA(18:1/30:1)-H	BUT1	−0.304	0.032
OAHFA(18:1/32:1)-H	BUT1	−0.303	0.032
OAHFA(18:1/30:1)-H	Meibum	0.295	0.037
LPC(18:1)+H	Meibum	0.294	0.039
TG(25:0/16:1/18:1)+NH4	Meibum	0.290	0.041
TG(16:1/18:1/18:2)+NH4	Meibum	0.290	0.041
TG(26:1/18:1/18:1)+NH4	Meibum	0.288	0.043
TG(16:0/18:2/20:5)+H	CR	0.287	0.043
OAHFA(18:1/30:1)-H	CR	0.283	0.046
TG(18:1/18:2/20:5)+H	Meibum	0.280	0.049

*Significant correlation between ocular lipid composition and ophthalmic clinical indexes in descending order*.

The results of correlation analysis between tear film lipid cluster components and ophthalmic clinical indexes showed that there was a significant correlation between SM and TH; TG and meibum; OAHFA and meibum, CR; LPC and meibum; and Cer and CR (all *p* < 0.05). Details can be found in Figure 6.

## Discussion

The finding of this study confirmed the therapeutic effect of IPL on MGD by symptoms and lipidomics analysis of the meibum. IPL treatment provides a novel approach that improves the management of MG diseases in a clinical setting, including improvement in the ocular surface health resulting from increasing tear production, stabilizing the tear film, improving corneal epithelial cell contacts, decreasing the conjunctival inflammation, and improving the quality and expressibility of the meibum from the MGs. Additionally, the lipids, such as LPC, OAHFA, TG, Cer, etc., were revealed alteration in patients with MGD after exposure to intense pulsed light (IPL) analyzed by lipidomics profiles. What is more, some of the lipids were highly related to the symptoms of MGD. These findings could be a valuable method to diagnose, prevent, and design personalized medical methods for MGD.

Intense pulsed light is a popular procedure for treating skin and ocular diseases. The mechanism of action of IPL systems is based on the principle of selective thermolysis, according to which certain targets, termed chromophores, are capable of absorbing and subsequently converting through nonradiative transfer the impinging light into heat energy. IPL treatment has been utilized for years in dermatological clinical practice (20, 21). Recently, this procedure was also applied in ophthalmology to treat MGD (22–24). Its use for this purpose stemmed from declines in DE symptoms noted in patients who underwent IPL treatment for facial rosacea. Previous research studies confirmed the efficacy of IPL treatment in relieving the symptoms and signs of MGD (23, 24). In our study, the patient's IPL treatment resulted in marked increases in the TBUT values. As previously reported, these rises occurred as a result of improved tear film stability and a reduction in the tear evaporation rate. These effects are in accord with our measurement of a significant increase in tear production. Furthermore, we found that the ocular surface health improved based on declines in bulbar redness and CF. All supported that IPL was performed as an effective treatment method for MGD.

It is understood that there is still a lack of specific reference data for evaluating the specific efficacy of IPL in the treatment of MGD. Through our study, we can quantitatively analyze the changes in lipid composition of the MG in patients with MGD, to infer its curative effect. We hypothesized that we could find the potential utility of novel biomarkers for predictive, preventive, and personalized medicine strategy for MGD through analysis of the alternation of the meibum lipidomics profile induced by IPL. The dysfunction of the MG was the initial factor of evaporative DE (9). Previous studies have demonstrated abnormal MGs, MG dropout, and abnormal keratosis in apolipoprotein E knockout mice. The corresponding microenvironmental cytokines all significantly changed. Studies showed that the increased expression of inflammatory factors such as IL-6 and TNF- α in acinar cells, the activation of NF- κ B signal pathway, and the enhancement of oxidative stress further simulated the histopathological, functional, and inflammatory changes in MGD (25). In addition, OAHFAs and their derivatives contribute to the formation of lipid polar gradients and participate in the connection between the tear lipid layer and the water layer to maintain tear film stability (26). LPC played an important role in maintaining the stability of tear film because of its chemical structure with both hydrophilic and lipophilic groups. These enzyme activities affected the regeneration of oxide membrane phospholipids and the maintenance of intracellular redox balance (27). Based on the effects of IPL treatment on the principal component analysis of patients with MGD, this procedure reversed several changes in the lipidomics profile toward patterns characteristic of those seen in healthy subjects. The moderate declines induced by IPL treatment in the MGD symptomology were associated with reversal of the hyperlipidemia in 12 of the 27 classes of lipids. It is possible that the restoration of the normal healthy profiles in this group of lipids contributed to the suppression of MGD symptomology (13). Of note, IPL treatment did not alter any of the other 15 classes of lipids changed by MGD. Furthermore, two sub classes (PC and PE) were higher than those in the untreated MGD group. Collectively, these results indicate that IPL treatment cannot completely reverse MGD symptomology, which is in accord with the failure of this procedure to fully reverse all of the changes in the lipidomics profile induced by MGD. Despite the reversal of the changes in an appreciable number of lipid classes by IPL treatment, there was an appreciable number of insensitive lipid classes that could underlie the failure of the treatment to fully reverse all of the MGD symptomologies. Nevertheless, we found that the increases in 13 subclasses of lipids in MGD could be reversed by IPL. They were rich in OAHFA, LPC, TG, and Cer. These lipids may be the suitable markers for monitoring responses to IPL treatment. TBUT, CR, and meibum quality are three clinical indices that were vindicated as being relevant markers to assess IPL treatment efficacy. In a word, the lipidomics profile can be regarded as a promised method to evaluate the effectiveness of treatment of MGD. What is more, the changes in OAHFA, LPC, TG, and Cer represented that the IPL can significantly change the composition of lipidomics profile in subjects, thus significantly improving the clinical symptoms of patients with MGD.

Except for the omega-3, one of OAHFAs, dietary supplementation, most of the treatment strategies for MGD were symptomatic treatment. However, it was controversial about omega-3 dietary supplementation (28, 29). What is more, OAHFAs were detected decreased in MGD in our results and other researchers reported (30). Lam et al. reported that OAHFA level was positively correlated with TBUT, reduction in eye evaporation rate, and degree of eye discomfort; LPC also had a similar trend and this is consistent with our study (31). The results of thin-layer chromatography analysis showed that the content of phosphatidylinositol in tears of patients with MGD decreased significantly, but increased significantly after IPL treatment, which coincided with our results, and our study further confirmed that there was a significant positive correlation between LPC and meibum, so effectively increasing the content of LPC lipid components in tear film can improve their ocular symptoms (32). Our research shows that there is a strong correlation between OAHFA, LPC, and ML, FL, ABUT and other clinical indicators. Emerging treatment options for MGD should be developed. Based on our results, from the changes in lipidomics profile, the decrease taken of OAHFAs, TGs and LPCs may be a benefit for treating MGD.

However, this study has potential limitations. First of all, the sample size of this study was small, and we would further expand the scale of the study to verify the reliability of our conclusions. Moreover, adolescents and children were not included in the study group. Further studies should expand the sample size to obtain data for all ages. In addition, we will do further basic research on how the changes in lipid composition before and after IPL treatment affect the pathological mechanism of MGD and observe how IPL regulates the signal pathway of lipid composition changes.

## Conclusions

In this study, we found that IPL treatment could reverse some of the MGD-induced hyperlipidemia. These changes alleviated some of the MGD symptomologies by stabilizing the tear film and improving the ocular surface health. Therefore, IPL can be used as an effective regimen for the treatment of MGD. The pertinent lipid changes include LPC, OAHFA, TG, Cer, etc. in patients with MGD can be used as the biomarkers for taking personalized medical methods for MGD.

## Data Availability Statement

The raw data supporting the conclusions of this article will be made available by the authors, without undue reservation.

## Ethics Statement

The studies involving human participants were reviewed and approved by Xinhua Hospital Affiliated to Medical College of Shanghai Jiao Tong University. The patients/participants provided their written informed consent to participate in this study.

## Author Contributions

HZ and S-NW carried out conceptualization. YS and L-YT involved in methodology. HZ, DX, and ZC provided resources. S-NW and JP performed visualization. HZ supervised the study. HZ contributed in funding acquisition. All authors have read and agreed to the published version of the manuscript.

## Funding

This study was supported in part by the Medicine & Engineering Collaboration Research Fund of Shanghai Jiao Tong University (ZH2018QNB27). The funders have no role in the study design, data collection and analysis, decision on publishing, or preparation of the manuscript.

## Conflict of Interest

The authors declare that the research was conducted in the absence of any commercial or financial relationships that could be construed as a potential conflict of interest.

## Publisher's Note

All claims expressed in this article are solely those of the authors and do not necessarily represent those of their affiliated organizations, or those of the publisher, the editors and the reviewers. Any product that may be evaluated in this article, or claim that may be made by its manufacturer, is not guaranteed or endorsed by the publisher.

## Abbreviations

IPL: intense pulsed light
MGD: meibomian gland dysfunction
T0: before treatment
T2: after treatment
TBUT: tear breakup time
MGE: meibomian gland expressibility
DE: dry eye
UHPLC-MS/MS: ultrahigh pressure liquid chromatography liquid chromatography-tandem mass spectrometry
CR: redness of conjunctival
CF: fluorescein staining
TG: triglyceride
Cer: ceramide
PC: phosphatidylcholine
SM: sphingomyelin
CerG1: simple Glc series G1
SO: sphingosine
LPC: lysophosphatidylcholine
LPE: lysophosphatidylethanolamine
MGDG: monogalactosyldiacylglycerol
PS: phosphatidylserine
DGDG: digalactosyldiacylglycerol
OAHFA: (O-acyl)-1-hydroxy fatty acid
PE: phosphatidylethanolamine
DGMG: digalactosylmonoacylglycerol
WE: wax ester
CerG2: simple Glc series G2
CO: coenzyme
LPG: lysophosphatidylglycerol
PG: phosphatidylglycerol
DG: diglyceride
LPI: lysophosphatidylinositol
LPS: lysophosphatidylserine
LSM: lysosphingomyelin
MGMG: monogalactosylmonoacylglycerol
PI: phosphatidylinositol
StE: stigmasterol ester
PCA: principal component analysis.
